# Supplementary material for: Predicting blood lead in Uruguayan children: Individual- vs neighborhood-level ensemble learners
Source: PLOS Glob Public Health. 2024 Sep 4;4(9):e0003607. doi: 10.1371/journal.pgph.0003607 (PMC11373808; doi:10.1371/journal.pgph.0003607)
Supplement: Attachment — Submitted filename: PGPH-D-23-01421_reviewer.pdf [file pgph.0003607.s002.pdf]

PLOS Global Public Health

Predicting Blood Lead in Uruguayan Children: Individual- vs Neighborhood-Level Ensemble Learners

--Manuscript Draft--

**Manuscript Number:**	PGPH-D-23-01421
**Article Type:**	Research Article
**Full Title:**	Predicting Blood Lead in Uruguayan Children: Individual- vs Neighborhood-Level Ensemble Learners
**Short Title:**	Predicting blood lead in Uruguayan Children
**Corresponding Author:**	Seth Frndak<br>University at Buffalo<br>Buffalo, UNITED STATES
**Order of Authors:**	Seth Frndak
	Elena I Queirolo
	Nelly Mañay
	Guan Yu
	Zia Ahmed
	Gabriel Barg
	Craig Colder
	Katarzyna Kordas
**Keywords:**	Machine Learning; lead; blood lead; neighborhood; prediction
**Abstract:**	Background: Predicting childhood blood lead levels (BLLs) has had mixed success, and it is unclear if individual- or neighborhood-level variables are most predictive.<br><br>Methods: We implemented an ensemble machine learning (ML) approach to identify the most relevant predictors of >2µg/dL BLL in urban children. A cross-sectional sample of 603 children (~7 years of age) recruited between 2009-2019 from Montevideo, Uruguay participated in the study. 77 individual- and 32 neighborhood-level variables were used to predict BLLs ≥2µg/dL. Three ensemble learners were created: one with individual-level predictors (Ensemble-I), one with neighborhood-level predictors (Ensemble-N), and one with both (Ensemble-All). Each ensemble learner comprised four base classifiers with 50% training, 25% validation, and 25% test datasets. Predictive performance of the three ensemble models was compared using area under the curve (AUC) for the receiver operating characteristic (ROC), precision, sensitivity, and specificity on the test dataset.<br><br>Results: Ensemble-I (AUC: 0.75, precision: 0.56, sensitivity: 0.79, specificity: 0.65) performed similarly to Ensemble-All (AUC: 0.75, precision: 0.63, sensitivity: 0.79, specificity: 0.69). Ensemble-N (AUC: 0.51, precision: 0.0, sensitivity: 0.0, specificity: 0.50) severely underperformed. Year of enrollment was most important in Ensemble-I and Ensemble-All, followed by household water Pb. Three neighborhood-level variables were among the top 10 important predictors in Ensemble-All (density of bus routes, dwellings with stream/other water source and distance to nearest river).<br><br>Conclusion: The individual-level only model performed best, although precision was improved when both neighborhood and individual-level variables were included. Future predictive models of lead exposure should consider proximal predictors (i.e., household characteristics).
**Additional Information:**	
**Question**	**Response**
**Financial Disclosure**	The results reported herein correspond to specific aims of grant R01ES023423 to

Powered by Editorial Manager® and ProduXion Manager® from Aries Systems Corporation

Enter a financial disclosure statement that describes the sources of funding for the work included in this submission and the role the funder(s) played. This includes grants and any commercial funding of the work or authors.<br><br>This statement will be typeset if the manuscript is accepted for publication.<br><br>*Please review the submission guidelines and the instructions link below for detailed requirements and guidance.*	investigator Katarzyna Kordas from the National Institute of Environmental Health Sciences. This work was also supported by grant(s) R21ES019949 and R21ES16523 from the National Institute of Environmental Health Sciences (PI: Kordas).
**Competing Interests**<br><br>On behalf of all authors, disclose any competing interests that could be perceived to bias this work.<br><br>This statement will be typeset if the manuscript is accepted for publication.<br><br>*Please review the instructions link below and PLOS Global Public Health's competing interests policy to determine what information must be disclosed at submission.*	No competing interests to declare.
**Data Availability**<br><br>Before publication, Authors are required to make fully available and without restriction all data underlying their findings. Please see our PLOS Data Policy page for detailed information on this policy.<br><br>A **Data Availability Statement**, detailing where the data can be accessed, is required at first submission. Insert your Data Availability Statement in the box	Data is available upon request to Dr. Katarzyna Kordas.

below.<br><br>Please see the data reporting section of our submission guidelines for instructions on what you need to include in your Data Availability Statement.<br><br>This statement will be typeset if the manuscript is accepted for publication.<br><br>*PLOS allows rare exemptions to address legal and ethical concerns. If you have legal or ethical restrictions, please use the box below to detail these in full sentences for the Journal team to consider.*	

Manuscript

Click here to access/download;Manuscript;Predicting BLLs 7.20.23.docx

Predicting Blood Lead in Uruguayan Children: Individual- vs Neighborhood-Level Ensemble Learners

Seth Frndak[1], Elena I. Queirolo[2], Nelly Mañay[3], Guan Yu[4], Zia Ahmed[5], Gabriel Barg[2], Craig Colder[4], Katarzyna Kordas[7]

[1]Department of Epidemiology: Johns Hopkins Bloomberg School of Public Health; [2]Department of Neuroscience and Learning, Catholic University of Uruguay, Montevideo, Uruguay; [3]Faculty of Chemistry, University of the Republic of Uruguay (UDELAR), Montevideo, Uruguay; [4]Department of Biostatistics: University of Pittsburgh USA; [5]Research and Education in eNergy, Environment and Water (RENEW) Institute University at Buffalo, The State University of New York USA; [6]Departament of Psychology: University at Buffalo, The State University of New York USA; [7]Departament of Epidemiology and Environmental Health: University at Buffalo, The State University of New York USA

Funding: The results reported herein correspond to specific aims of grant R01ES023423 to investigator Katarzyna Kordas from the National Institute of Environmental Health Sciences. This work was also supported by grant(s) R21ES019949 and R21ES16523 from the National Institute of Environmental Health Sciences (PI: Kordas).

Correspondence:

Dr. Seth Frndak
Department of Epidemiology
University at Buffalo School of Public Health and Health Professions
+1 716 795-7377 (phone)
sethfrnd@buffalo.edu (email)

Abstract

Background: Predicting childhood blood lead levels (BLLs) has had mixed success, and it is unclear if individual- or neighborhood-level variables are most predictive.

Methods: We implemented an ensemble machine learning (ML) approach to identify the most relevant predictors of >2µg/dL BLL in urban children. A cross-sectional sample of 603 children (~7 years of age) recruited between 2009-2019 from Montevideo, Uruguay participated in the study. 77 individual- and 32 neighborhood-level variables were used to predict BLLs ≥2µg/dL. Three ensemble learners were created: one with individual-level predictors (Ensemble-I), one with neighborhood-level predictors (Ensemble-N), and one with both (Ensemble-All). Each ensemble learner comprised four base classifiers with 50% training, 25% validation, and 25% test datasets. Predictive performance of the three ensemble models was compared using area under the curve (AUC) for the receiver operating characteristic (ROC), precision, sensitivity, and specificity on the test dataset.

Results: Ensemble-I (AUC: 0.75, precision: 0.56, sensitivity: 0.79, specificity: 0.65) performed similarly to Ensemble-All (AUC: 0.75, precision: 0.63, sensitivity: 0.79, specificity: 0.69). Ensemble-N (AUC: 0.51, precision: 0.0, sensitivity: 0.0, specificity: 0.50) severely underperformed. Year of enrollment was most important in Ensemble-I and Ensemble-All, followed by household water Pb. Three neighborhood-level variables were among the top 10 important predictors in Ensemble-All (density of bus routes, dwellings with stream/other water source and distance to nearest river).

Conclusion: The individual-level only model performed best, although precision was improved when both neighborhood and individual-level variables were included. Future predictive models of lead exposure should consider proximal predictors (i.e., household characteristics).

Keywords: Machine learning, low blood lead levels, neighborhood, prediction

1.0 Introduction

Children's blood lead levels (BLLs) have continued to decline worldwide. Among U.S. children <6 years of age, mean BLLs decreased from 13.7µg/dL in 1976-1980 (1) to 1.3µg/dL in 2007-2010 (2). Recent estimates report a mean of 0.80µg/dL among children participating in the 2011-16 National Health and Nutrition Examination Survey (NHANES) (3). Despite this promising trend, 1 in 3 children (~800 million) worldwide are estimated to have BLLs ≥5µg/dL (4). Low lead levels are still concerning in children as declines in IQ are found at BLLs <10µg/dL (5, 6). Recently, the Centers for Disease Control lowered the actionable threshold from 5 to 3.5µg/dL (7). As no safe threshold of lead exposure has been found, prevention of exposure is of public health concern. Identification of high-risk children using predictive modeling could be an important preventive tool.

Predicting geographic areas with a high prevalence of lead-exposed children (8), may be useful for spatially targeted public health interventions. However, individual-level predictive models could also be implemented in clinical settings where testing is limited (9), or when there is suspected continued exposure beyond the age-required testing window (1-2 years in the US). Numerous efforts have been made to predict children's BLLs with questionnaire-based screening tools, but with limited success. A 2019 review of lead screening questionnaires revealed a wide range of success predicting BLLs >10 µg/dL. Authors of the review concluded that screening questionnaires "are not accurate for identifying children with elevated blood lead levels" (10). An alternative method of predicting BLLs is through machine learning (ML) classifiers. Recently, Potash et al. (2020) demonstrated that random forest (RF) outperformed logistic regression in predicting BLLs ≥6 µg/dL (11). Liu et al. (2021) used an ensemble ML approach to predict BLL in Australian children <5 years of age (12, 13). While promising, these predictive ML models do have limitations. Potash et al. 2020, for example, used a high cut-off for lead exposure while Liu et al. 2021 included data from 1991-2015. ML is promising, but predictive models must be updated to predict lower levels of exposure and include more recent data to reflect global declines in child lead levels.

While ML can handle high-dimensional data, more parsimonious models are easier to deploy, and are less likely to be overfit to irrelevant predictors. For example, while neighborhood-level variables are easier to generate and associate with individual child BLLs, they may add noise to models that could benefit from parsimony. Potash et al. (2020) used only neighborhood-level variables (11), while Liu et al. (2021) used both individual- and neighborhood-level predictors. Future predictive models of low-level lead exposure at the individual level might benefit from more focused data inputs. Furthermore, predictive models can be useful in countries where pediatric lead testing is limited. To implement such models, we must test if individual-, neighborhood-level, or a combination of predictors is best for model development.

Leveraging BLLs of ~7 year-old children from Montevideo, Uruguay, this study aimed to: 1) develop a ML model predictive of BLLs ≥2 µg/dL, and 2) identify whether individual, neighborhood or a combination are most relevant for predicting low-level lead exposure. This knowledge will help inform development of datasets and predictive models that can be used for lead screening.

2.0 Materials and Methods

2.1 Sample

Salud Ambiental Montevideo (SAM) is an ongoing cohort of children residing in the city of Montevideo, Uruguay. Children were recruited as first-graders (~7 years of age) between July 23rd, 2009 and June 24th, 2019 from several elementary schools located in areas of suspected toxic metal exposure. Recruitment procedures for children enrolled in 2009-13 can be found elsewhere (14). Two changes for years 2015-19 should be noted while all other protocols stayed unchanged: 1) the study received approval from the Ministry of Education to recruit children from public elementary schools, 2) study assessments, including blood draws, took place at the SAM research suite at the Catholic University of Uruguay as opposed to children's schools. For this study, we excluded 97 children without blood lead measurements, 17 children without information on household location or residing outside the city limits of Montevideo. Furthermore, as our limit of detection (LOD) for

blood lead measurement improved during data collection, we removed observations with analytical LOD ≥2 µg/dL (n=139). This resulted in an analytical sample of 603. Study protocols were approved by the institutional review boards of the University at Buffalo, the University of the Republic of Uruguay, and the Catholic University of Uruguay. All caregivers gave their written consent for participation and publication. Children provided assent. Participants were not involved in the design or dissemination of this research.

2.2 Blood Lead Measurements

A trained phlebotomist collected blood samples from children between 8 and 11am, after an overnight fast. Samples were subsequently transported on ice for measurement at the Toxicology Laboratory at the University of the Republic of Uruguay with Atomic Absorption Spectrometry (AAS). In our analytical sample, 5% were analyzed using flame ionization (AAS, VARIAN SpectrAA-55B - limit of detection (LOD) 1.8 µg/dL) during the early enrollment period (2009-2013). Throughout enrollment, graphite furnace AAS was also used (GFAAS/Thermo ICC 3400 – LOD 1, 24%, 2015-2016 & 0.4 µg/dL, 48%, 2016-2019) (GFAAS/Varian Spectra AA55B – LOD 0.8, 23%, 2010-2013). Laboratory protocols followed standard procedures (15).

2.3 Individual-Level Predictors

We selected 110 predictors *a priori* based on previous literature of lead exposure in children. A complete list of these variables is presented in **Table 1**. Among those, 77 were measured at the individual level, representing household water testing, clinical, nutritional and questionnaire measures. We included two household water metals: water lead (Pb) and water iron (Fe). Household water was sampled from the kitchen in a 100ml plastic container. Water samples were transported to Pennsylvania State University and analyzed using Inductively Coupled Plasma Mass Spectrometry (ICP-MS) with Collision Cell Technology (Thermo Scientific XSERIES2, Bremen, Germany). More detail on water sample collection and analysis is available in previous publications (16). Four clinical measures were included: body mass index (BMI), height for age z-score (HAZ), weight for age z-score (WAZ), and blood hemoglobin (g/dL). A nurse or nutritionist took three repeated measures of the child's height and weight. The average height and weight were used to calculate the child's BMI and HAZ. Hemoglobin was measured in a drop of blood using a hemoglobinometer (HemoCue, Lake Forest, CA). The 10 dietary measures were collected using two 24-hour dietary recalls administered at least two weeks apart. A trained nutritionist administered the recall to the caregiver, with the child present to aid in recollection; one recall was administered in person and the second by phone. A database of 342 typical Uruguayan foods was used to calculate dietary kilocalories, iron (mg/day), protein (g/day), fat (g/day), fiber (g/day), carbohydrates (g/day), zinc (mg/day), calcium (mg/day), folate (mg/day), and vitamin C (mg/day) for each of the two days, and an average was calculated. We also created 25 food groups (g/day) based on individual foods as described previously (17). Finally, 36 individual-level variables (**Table 1**) were obtained via questionnaire and completed by the caregiver, who answered questions about family socioeconomic status, health history, and household cleaning practices. These included index scores such as number of possessions of wealth and the Home Observation for the Measurement of the Environment Inventory (HOME). The number of possessions of wealth was calculated based on ownership of 12 household assets. Exploratory factor analysis (EFA) retained a single factor with 5 possessions, including DVD player, computer, car, washing machine, and landline phone. The HOME inventory was completed by a social worker during a visit to the child's home. A summary measure of the home environment was calculated (HOME Inventory) (18), with higher scores reflecting greater household enrichment.

2.4 Neighborhood-Level Predictors

The remaining 32 variables were collected at the neighborhood-level, based on household location. These variables can be categorized into census segment characteristics, NDVI and distance/density measures.

Statistical software R was used to calculate these variables using several packages including *rgeos* (19), *rgdal* (20), *sp* (21), *RStoolbox* (22), and *raster* (23).

Aggregated 2011 census segment characteristics were downloaded from the Intendencia de Montevideo Servicio de Geomática (IMSG) (24). Participants were assigned aggregated census segment characteristics based on the census segment within which their home was located. Additional information on the census segment database used for our analysis has been previously published (25). Census segment variables included a neighborhood disadvantage factor; square area of the census segment (meters^2); number of persons residing in the census segment, percentage of households in the census segment with 1) city network water source, 2) well water source, and 3) stream or other water source; and percentage of households in the census segment with 1) city network waste disposal, 2) septic tank waste disposal, and 3) stream or other waste disposal.

A multi-date, 150-meter and 300-meter buffer NDVI score was calculated based on 4-band, 3-meter raster files derived from Planet Image (26). Cloud-free satellite images of Montevideo were downloaded for 6 summer-time dates: December 24, 2018; January 28, 2019; February 19, 2019; December 23, 2019; January 28, 2020; and February 23, 2020. High-resolution data (3-meters) is only available after 2017. NDVI was calculated for each 3-meter raster, on each date, with the standard formula: NDVI = (near infrared band – red band) / (near infrared band + red band). Average area NDVI was then calculated for each date using a 150-meter buffer around the participant's home. Finally, an overall, average NDVI buffer score was calculated for each participant across the 6 dates.

Several distance measures (in meters) were created from IMSG data. Locations and boundaries of informal settlements were determined by the Programa de Integración de Asentamientos Irregulares (PIAI). An irregular settlement was defined as an area with 10 or more dwellings built without authorization or permit. Locations and boundaries of public greenspaces (i.e., parks, gardens, landscaped areas) were determined using photogrammetry of aerial photos by the IMSG. A list of industries provided by the IMSG was reduced to only include those with potential for environmental contamination with toxic elements. These 123 industries included: motor vehicle manufacturers, manufacturers of metallic products, and manufacturers of paints and plasticizers. Geolocations of 163 local ferias – open air markets – were gathered by local partners in Montevideo. These ferias are often a source of fresh produce. The IMSG also provides locations of all registered roads, bus routes, waterways, and major rivers in Montevideo. We calculated the distance (in meters) to the nearest bus route. Waterways and rivers within the city of Montevideo were downloaded from the IMSG. The IMSG identified waterways and rivers using photogrammetry of aerial photos. Rivers were differentiated from waterways by the IMSG based on flow rate.

Densities of ferias, roads, and bus routes around the participant's home were also calculated. We calculated the total length in meters for roads, bus routes, and waterways within 500- and 1,000-meter circular buffers around the participant's home. Density was then calculated as the total length divided by the square area (meters^2) of each circular buffer. We did not calculate the density of rivers due to the overall low density of rivers in Montevideo. The density of markets was calculated as the number of ferias within 1-kilometer and 500-meter buffers. The IMSG also maintains locations of gas stations (ANCAP, ESSO, and Petrobras) in the city of Montevideo. We counted the number of gas stations within 1-kilometer and 500-meter buffers as a measure of density. Locations of special containers for disposal of waste including batteries, cans, plastics, and glass were also counted within 500- and 1,000-meter buffers.

2.5 Missing Data Imputation

We imputed missing values using the *missForest* package in R (27). *missForest* is favorable compared to other imputation techniques (28) and useful when unable to account for multiple imputed datasets. Random forest for missing data is also robust when predictors have heavy missingness (>75%), as is the case with some of our predictors (29).

The number of trees (*ntree*) was set to 500 and the number of variables at each node (mtry) was set to 40. This was decided after an iterative process of first testing an increasing number of *mtry* (from 2 to 50) and then testing an increasing number of *ntree* (100 to 1,000 stepped by 100). The final Normalized Root-Mean-Square Error (NRMSE) was 0.001% (a measure of imputation error for continuous variables). The Percent Falsely Classified (PFC) was 20.6% (a measure of imputation error for discrete variables).

2.6 Generation of Three Ensemble Models

Three ensemble models were generated using different combinations of predictors. Three base classifiers were chosen: generalized linear model (GLM) with an elastic net penalty, random forest (RF), gradient boosting machine (GBM), and deep neural network (DNN). GLM with an elastic net penalty combines the advantages of both the Least Absolute Shrinkage and Selection Operator (LASSO) and Ridge regression to find the optimal penalty factor, shrinking the beta-coefficient of predictors to zero if not associated with the outcome (LASSO) or near zero (Ridge). RF is an aggregation of randomly generated decision trees, useful in classification and non-parametric estimation of regression problems. GBM is an extension of RF which aggregates decision trees with fewer branches, using classification performance to inform subsequent decision trees. DNN uses neural networks to optimize prediction forward and back-propagation to weight nodes based on classification performance. A stacked ensemble is a method of combining the predictions of all base classifiers using a meta-GLM, improving classification performance and preventing over-fitting. Further discussion of these algorithms and stacking methods can be found in James et. al. 2013 (30).

An individual-level only ensemble was estimated using only the 77 individual-level predictors (Ensemble-I). A neighborhood-level only ensemble was estimated using only the 32 neighborhood-level predictors (Ensemble-N). Lastly, all 109 predictors were used to estimate Ensemble-All. The following process was repeated for each of the three ensembles using the same random seed to ensure data splitting was replicated across combinations of predictors.

First, we created three randomly split training (50%), validation (25%), and test (25%) datasets. Four base classifiers were used to create the ensemble: a generalized linear model with the elastic net penalty (GLM), random forest (RF) (31), gradient boosting machine (GBM) (32), and a deep neural network (33, 34). Optimization and final selection of the hyper-parameters for each base learner was based on the greatest area under the curve (AUC) for the receiver operating characteristic (ROC). Model calibration for all base learners was performed using a grid search for optimal hyper-parameters with 10-fold cross validation on the training dataset. Following the example laid out by Ahmed et al. 2021 (35), we set 0.001 as the stopping tolerance and 2 as the stopping rounds. Stopping tolerances help prevent overfitting, improving overall classification. We also report the hyperparameters for each base model in our results as in Ahmed et al. 2021. The best-performing base learner based on highest AUC in the validation dataset was subsequently incorporated into a stacked ensemble. Stacking creates a meta-dataset containing the predictions of each base learner and uses a subsequent meta-classifier to create an ensemble prediction (36). We chose a generalized linear model (GLM) as our meta-classifier for the stacked ensemble. Tuning of the stacked ensemble was performed using 10-fold cross-validation. Model creation was implemented using the *h2o* package in R (37).

2.6 Model Performance Metrics

Performance of the final three ensembles (Ensemble-I, Ensemble-N, and Ensemble-All) was assessed using AUC, AUC-PR precision, sensitivity, and specificity in the test dataset. AUC ranges from 0 to 1 and is based on the ROC curve. An AUC of 1 indicates perfect classification. The other performance metrics were calculated from the confusion matrix of predicted classifications based on the F1-optimal threshold. The confusion matrix is a 2x2 table of classifications including true positives (TP), false positives (FP), false negatives (FN) and true negatives (TN). Formula for the F1 score (37) is:

$$F1 = 2\,(\frac{(precision)(sensitivity)}{precision + sensitivity})$$

Where precision is defined as TP/(TP+FP) and sensitivity is TP/(TP+FN). F1 scores range from 0 to 1, with 1 indicating all positives and negatives were correctly classified. Specificity is calculated as TN / (FP+TN), and when high is meant to optimize true detection of a non-event.

2.7 Variable Importance

We implemented explainable ML using the *DALEX* package in R to derive variable importance with the test dataset (38). First, permutation-based calculations were used to estimate variable importance, which reflects the classification performance of the learner if that variable was removed. This method randomly reorders the variable of interest, recalculates the prediction, and the drop in learner performance is recorded (dropout loss). Permutation-based variable importance can be useful when comparing variable importance across many learners as in an ensemble (12). Variable importance statistics were calculated using 1 - Area Under the Curve (AUC) as the dropout loss function.

3.0 Results

3.1 Sample Characteristics

A complete list of variables with sample characteristics is presented in **Table1**. Children were ~7 years of age on average, and evenly distributed by sex (52% male). Recruitment varied slightly over time from 2010 to 2019 (range 5%-18%), with the most children recruited in 2018. Children had low to average BMI (M = 16.7, SD = 2.6 kg/m^2) and hemoglobin level indicative of absence of anemia (M = 13.2, SD = 0.9 g/dL). Many children came from homes of low socioeconomic status with a low number of possessions of wealth (M = 2.8, SD = 1.3), and ~23% of households with monthly income < $10,000 Uruguayan Pesos (~$370.00 USD in June 2015) (39).

Children in our sample came from more disadvantaged neighborhoods (mean = 1.5, SD = 1.1) with a neighborhood disadvantage score around one standard deviation above the city mean of 0. On average, children lived within 150 meters of a bus route (mean = 143.6, SD = 129.0), within 400 meters of a waterway (M = 360.0, SD = 446.1), and within 1 kilometer of an industry using in toxic metals in production (M = 937.4, SD = 608.1). Children also lived, on average, within 300 meters of greenspace (M = 246.3, SD = 208.4) and average 150-meter buffer NDVI (M = 0.3, SD = 0.1). Close to half of children had BLLs ≥2 µg/dL (47%).

3.2 Ensemble Performance

We report model performance during training, validation and testing for all base classifiers and the stacked ensemble in **Table 2**.

3.2.1 Ensemble-I

Hyperparameters for the best base classifiers in Ensemble-I: (GLM (AUC test = 0.61): alpha of 0.0, lambda of 2.54, RF (AUC test = 0.72): 50 trees, column sample rate of 0.59, max depth of 20; GBM (AUC test = 0.75): 100 trees, column sample rate of 0.40, max depth of 2, 1 minimum number of rows; DNN (AUC test = 0.68): 3 hidden layers with 100 units, a “Maxout” activation function). ROC curves for Ensemble-I and all base classifiers on the test dataset are presented in **Figure 1 (Panel A)**. AUC in the test dataset for Ensemble-I was 0.75. At the F1-optimal threshold, precision was 0.56, sensitivity 0.79, specificity 0.65.

Variable importance for Ensemble-I is presented in **Figure 2 (Panel A)**. Top ten predictors included: year of enrollment, household water Pb (ppm), HOME inventory Score, household water Fe (ppm), height for age z-score, fruit consumption (g/d), type of preschool attended, household crowding, child age in months, money spent on food last month.

3.2.2 Ensemble-N

Hyperparameters for the base classifiers in Ensemble-N: (GLM (AUC test = 0.49): alpha of 0.69, lambda of 0.1, RF (AUC test = 0.53): 4,150 trees, column sample rate of 0.19, max depth of 40; GBM (AUC test = 0.59): 1,000 trees, column sample rate of 0.80, max depth of 18, 100 minimum number of rows; DNN (AUC test = 0.49): 4 hidden layers with 10 units, a "Tanh" activation function). ROC curves for Ensemble-N and all base classifiers are presented in **Figure 1 (Panel B)**. AUC for Ensemble-I was 0.51. At the F1-optimal threshold, precision was 0.0, sensitivity 0.0, specificity 0.50.

Variable importance for Ensemble-N is presented in **Figure 2 (Panel B)**. Top ten predictors included: percent dwellings that dispose waste into the city network, percent dwellings with city network water source, distance to nearest feria (m), density of bus routes (1km buffer), distance to nearest irregular settlement (m), density of all registered roads (1km buffer), distance to nearest gas station (m), distance to nearest greenspace (m), density of waterways (1km buffer), and percent of dwellings with a stream or other water source.

3.2.3 Ensemble-All

Hyperparameters for the base classifiers in Ensemble-All: (GLM (AUC test = 0.57): alpha of 0.0, lambda of 4.80, RF (AUC test = 0.73): 310 trees, column sample rate of 0.68, max depth of 50; GBM (AUC test = 0.70): 1,000 trees, column sample rate of 0.80, max depth of 18, 100 minimum number of rows; DNN (AUC test = 0.61): 3 hidden layers with 50 units, a "Maxout" activation function). ROC curves for Ensemble-All and all base classifiers are presented in **Figure 1 (Panel C)**. AUC for Ensemble-All was 0.75. At the F1-optimal threshold, precision was 0.63, sensitivity 0.79, specificity 0.69.

Variable importance for Ensemble-All is presented in **Figure 2 (Panel C)**. Top ten predictors included: year of enrollment, household water Pb (ppm), density of bus routes (1km buffer), HOME Inventory score, fruit consumption, yogurt consumption, percent dwellings with stream or other water source, distance to the nearest river (m), dark leafy vegetable consumption (g/d), and household crowding.

4.0 Discussion

Even at relatively low levels, lead can impact children's cognition and behavior. Thus, predicting which children may have BLLs ≥2µg/dL using recently collected data is of public health importance. However, it is unclear what combination of variables (individual-level, neighborhood-level, or both) are best for BLL prediction. We used three different combinations of predictors when generating our ensemble classifiers: individual-level only predictors (Ensemble-I), neighborhood-level only predictors (Ensemble-N) and both types of predictors (Ensemble-All). Overall, Ensemble-I and Ensemble-All had similar AUC in the test dataset within two decimal places (0.75), followed by Ensemble-N (0.51). The severe underperformance of Ensemble-N suggests that individual-level predictors may perform better, or we did not successfully capture an important neighborhood-level predictor. While Ensemble-All and Ensemble-I performed similarly, Ensemble-All did have better precision (0.63 vs 0.56, respectively). While studies have shown promising predictive power based exclusively on neighborhood-level predictors, our study suggests that using either individual-only or both individual- and neighborhood-level variables performs best for predicting BLLs ≥2µg/dL. Collecting more proximal predictors (i.e., household water lead and diet) may be best when generating predictive models of low-level lead exposure.

In Ensemble-I, socioeconomic status, diet, and physical dwelling characteristics were most important for prediction. In general, lower socioeconomic status is directly associated with BLLs in children (40, 41). Diet is also associated with BLLs in children and may be especially relevant for low-level exposure (42). Specifically, fruit consumption was important for prediction. Especially citrus are rich in vitamin C which has been theorized to be associated with lower blood lead levels (43), although clinical trials have been inconclusive

(44). Finally, household water lead, HOME inventory score, and crowding, all associated with housing quality, may be associated with BLLs in children (45). Curation of data that includes proximal variables such as diet, household characteristics and socioeconomics may enhance low-level BLL prediction.

In Ensemble-I and Ensemble-All year of enrollment was the most important predictor. We know BLLs are decreasing in children worldwide and it is not uncommon to note secular trends in children's BLLs. Liu and colleges (Australia) reported year of enrollment as the most important predictor of BLLs in Australian children (12). In a recent publication that used ML Bayesian networks to relate water lead and BLL in North Carolina, US, year of blood draw was also the most important predictor of child BLLs ≥ 2 μg/dL (46). It is difficult to infer from our data what may be changing in the environment across years. First, we were unable in this cross-sectional study to evaluate changes in BLLs of our children over time. Second, neighborhood-level variables were collected at a single time point, thus we cannot evaluate how changes in the neighborhood environment may be related to BLL over time. We plan to investigate secular trends in BLLs and participant characteristics in future studies. To further probe our results, we added year of enrollment to the Ensemble-N variable set. While predictive power was improved, Ensemble-N still did not perform as well as Ensemble-I or Ensemble-N.

We note that some caution is warranted in interpreting these findings. First, the most important predictor in Ensemble-I was household water Pb. We previously published that household water Pb was only minimally associated with urinary and blood lead levels in our sample, dependent on children's iron status (16). We note that the analyses in Ravenscroft et al. 2018, were causal in nature, adjusting for socioeconomic status indicators, thereby obtaining the effect estimates adjusted for confounding. In ML, variable importance cannot be interpreted in the same manner as effect estimates in causal models. Complex interactions between predictors can affect overall importance (47, 48). It should also be noted that precision (or positive predictive value) was low across models (Ensemble-I precision 0.56), while sensitivity (or negative predictive value) was high (Ensemble-I sensitivity 0.79). Thus, our ability to rule out children without BLLs ≥2μg/dL was strong, but positive prediction of BLLs ≥2μg/dL was poor. This further suggests that our previous work was correct in asserting that household water Pb, and red meat consumption were not strongly associated with BLL, and our predictive model is likely using these predictors to correctly classify children with BLLs <2μg/dL.

There are additional limitations in our study. First, we only considered four popular, high-performing base classifiers. The chosen classifiers may not fit our data well and therefore, some important predictors may not have been selected. We did not include all potentially relevant predictors. For example, soil lead levels are known to contribute to children's BLLs (49). It is important to note that predictive models utilizing area-level characteristics will necessarily be context specific and may not translate well to other samples. Geographic and neighborhood predictors may be more relevant in the U.S. where geographic segregation has contributed to BLLs in U.S. children (50). Lastly, some of our variables, including household water iron and water lead had a high number of missingness. Our model may have been improved with less missing data.

As for strengths, this is the first study to attempt ensemble ML-based prediction of child BLLs ≥2μg/dL with a large set of individual- and neighborhood-level variables. In total, we had 109 variables (77 individual- and 32 neighborhood-level). We also included uncommonly used variables such as food and nutrient consumption, and home cleaning practices. Furthermore, such sophisticated predictive models are underemployed for predicting toxicant exposure in the Global South. Lastly, the AUC for Ensemble-I was acceptable, though predictive performance could be enhanced.

5.0 Conclusions

In summary, an ensemble ML with only neighborhood-level variables severely underperformed. Ensembles with individual-level variables or a combination of both is ideal. Future predictive models in global contexts may learn from our findings by including more proximal predictors of socioeconomics, diet, and household characteristics.

393

Table 1. Sample characteristics for all variables and number missing (n=603)

Domains and Variables	Descriptive Statistics	Number Missing
**Individual Level Measures**		
Blood Lead Level ≥2µg/dL (%)	47.1%	0
Year of Enrollment		0
2010	2.3%	
2011	4.3%	
2012	10.5%	
2013	11.3%	
2015	10.6%	
2016	17.1%	
2017	17.7%	
2018	21.7%	
2019	4.5%	
Child Sex (% Male)	51.9%	0
Child Age in Months (Mean ± SD)	82.3 (6.08)	1
Serum Hemoglobin (g/dL) (Mean ± SD)	13.2 (0.9)	4
Child's BMI kg/m^2 *(Mean ± SD)*	16.8 (2.6)	7
Child's Height for Age Z-Score (Mean ± SD)		
Child's Weight for Age Z-Score (Mean ± SD)		
Household Water Iron (Fe) Content (ppm)	73.7 (363.3)	456
Household Water Lead (Pb) Content (ppm)	2.5 (4.8)	468
Number of Siblings (Mean ± SD)	1.8 (1.6)	41
Parental Report of Anemia (% Yes)	8.8%	43
Parental Report – Child Hospitalized Ever (% Yes)	45.7%	38
Parental Report – Child Taken Iron Supplements Last 2 Months (% Yes)	4.6%	37
Mother's Education in Years (Mean ± SD)	8.3 (2.4)	14
Father's Education		72
Any Primary Education	38.6%	
Any Secondary Education	50.1%	
Any Bachelor's or Greater	11.3%	
Mother's Age in Years (Mean ± SD)	33.5 (7.02)	22
Father's Age in Years (Mean ± SD)	36.3 (8.06)	72
Mother is Employed (% Yes)	59.8%	86
Father is Employed (% Yes)	87.6%	184
Either Caregiver Smokes (% Yes)	62.8%	28
Average Number of Cigarettes the Mother Smokes per Day (Mean ± SD)	3.9 (7.2)	45
Average Number of Cigarettes the Father Smokes per Day (Mean ± SD)	5.6 (8.8)	127
Times per Month Mother Smoked During Child's Pregnancy		53
Never (%)	78.2%	
1-2 per day (%)	4.9%	
3-4 per day (%)	5.8%	
5+ per day (%)	11.1%	
Parent Report Hours per Week Child Plays Outside during Summer	8.0 (7.2)	60
Type of Preschool Attended		27
Private	35.9%	
Public	61.1%	
Did not Attend	3.0%	
Number of Possessions of Wealth (Mean ± SD)	2.8 (1.3)	31

Money Spent on Food for the Household Last Month (% <$3,000 UYU)	88.1%	99
Household is Crowded (>2 Persons per Room) (% Yes)	33.9%	29
Year House was Built (Mean ± SD)	1983 (116.2)	316
Family Members Remove Shoes When Entering House (% Yes)	6.8%	31
Kids <5 Years in the Household (% Yes)	41.6%	0
Cooking Water is Filtered (% Yes)	9.3%	33
Use Filtered Drinking Water (% Yes)	45.0%	30
Family Cultivates or Consumes Food from a Garden? (% Yes)	14.6%	33
Either Parent Works in a Job with Source of Exposure (% Yes)	33.3%	3
How Many Years Ago Did You Last Paint the House Inside?		72
< 6 Months (% Yes)	21.9%	
≥ 6 Months - < 1 Year (% Yes)	10.6%	
≥ 1 Year - < 3 Years (% Yes)	45.8%	
3+ Years (% Yes)	15.8%	
Never Painted (% Yes)	6.0%	
Windows are Open >5 Hours a Day in Summer (% Yes)	63.1%	29
Windows are Open >5 Hours a Day in Winter (% Yes)	19.3%	28
Floors Cleaned > 1 Time per Week (% Yes)	91.5%	28
Dusting > 1 Time per Week (% Yes)	69.5%	32
Household Entryways have a Doormat (% Yes)	50.5%	33
HOME Inventory Score (Mean ± SD)	40.3 (10.2)	94
Household Monthly Income		73
< $10,000 UYU	22.8%	
$10,000 - $14,999 UYU	23.4%	
$15,000 - $24,999 UYU	26.0%	
$25,000 + UYU	27.7%	
Estimated Total Kilocalories	2040.6 (592.9)	9
Estimated Dietary Iron (mg/d) (Mean ± SD)	9.4 (4.3)	9
Estimated Dietary Protein (g/d) (Mean ± SD)	67.0 (19.1)	9
Estimated Dietary Fat (g/d) (Mean ± SD)	65.3 (24.1)	9
Estimated Dietary Carbohydrates (g/d) (Mean ± SD)	297.2 (101.7)	9
Estimated Dietary Fiber (g/d) (Mean ± SD)	3.5 (2.3)	9
Estimated Dietary Zinc (mg/d) (Mean ± SD)	4.8 (2.1)	9
Estimated Dietary Calcium (mg/d) (Mean ± SD)	656.0 (246.8)	9
Estimated Dietary Folate (mg/d) (Mean ± SD)	451.2 (197.4)	9
Estimated Dietary Vitamin C (mg/d) (Mean ± SD)	49.1 (49.3)	9
24-Hour Recall of Fruit Consumption (g/d) (Mean ± SD)	106.2 (119.0)	9
24-Hour Recall of Dark Leafy Vegetable Consumption (g/d) (Mean ± SD)	0.80 (2.9)	9
24-Hour Recall of Red Orange Vegetable Consumption (g/d) (Mean ± SD)	8.8 (21.7)	9
24-Hour Recall of Beans and Peas Consumption (g/d) (Mean ± SD)	2.0 (9.1)	9
24-Hour Recall of Other Vegetables Consumption (g/d) (Mean ± SD)	8.5 (34.9)	9
24-Hour Recall of Potato Consumption (g/d) (Mean ± SD)	24.2 (42.9)	9
24-Hour Recall of Bread Consumption (g/d) (Mean ± SD)	43.7 (49.4)	9
24-Hour Recall of Grain Consumption (g/d) (Mean ± SD)	27.5 (38.6)	9
24-Hour Recall of Pasta Consumption (g/d) (Mean ± SD)	26.6 (41.5)	9
24-Hour Recall of Red Meat Consumption (g/d) (Mean ± SD)	35.2 (48.1)	9
24-Hour Recall of White Meat Consumption (g/d) (Mean ± SD)	16.7 (36.7)	9
24-Hour Recall of Processed Meat Consumption (g/d) (Mean ± SD)	45.6 (59.6)	9
24-Hour Recall of Egg Consumption (g/d) (Mean ± SD)	6.5 (11.8)	9
24-Hour Recall of Milk Consumption (g/d) (Mean ± SD)	274.7 (189.1)	9
24-Hour Recall of Cheese Consumption (g/d) (Mean ± SD)	5.3 (12.2)	9

24-Hour Recall of Yogurt Consumption (g/d) (Mean ± SD)	54.6 (91.1)	9
24-Hour Recall of Soy Product Consumption (g/d) (Mean ± SD)	25.4 (63.8)	9
24-Hour Recall of Fat and Oil Consumption (g/d) (Mean ± SD)	8.5 (8.8)	9
24-Hour Recall of Milk-Based Dessert Consumption (g/d) (Mean ± SD)	18.8 (36.2)	9
24-Hour Recall of Sweets Consumption (g/d) (Mean ± SD)	40.2 (34.0)	9
24-Hour Recall of Pastries Consumption (g/d) (Mean ± SD)	65.4 (56.4)	9
24-Hour Recall of Fried Potatoes Consumption (g/d) (Mean ± SD)	10.2 (25.1)	9
24-Hour Recall of Sweetened Beverages Consumption (g/d) (Mean ± SD)	310.9 (289.0)	9
24-Hour Recall of Pizza or Dinner Pie Consumption (g/d) (Mean ± SD)	110.1 (121.4)	9
24-Hour Recall of Sauces Consumption (g/d) (Mean ± SD)	3.9 (14.2)	9
**Neighborhood Level Measures**		
Neighborhood Disadvantage (Mean ± SD)	1.5 (1.1)	0
Square Area (m) in Census Segment Where Child Lives (Mean ± SD)	601,469.7 (934,140.6)	0
Number of Persons in Census Segment Where Child Lives (Mean ± SD)	2,230.6 (1274.8)	0
Normalized Difference Vegetation Index (NDVI) 150m Buffer (Mean ± SD)	0.3 (0.1)	0
Normalized Difference Vegetation Index (NDVI) 300m Buffer (Mean ± SD)	0.3 (0.1)	0
Distance to Nearest Informal Settlement (m) (Mean ± SD)	226.0 (400.3)	0
Distance to Nearest Greenspace (m) (Mean ± SD)	246.3 (208.4)	0
Distance to Nearest Feria (m) (Mean ± SD)	829.9 (464.9)	0
Number of Markets within 1km Buffer (Mean ± SD)	1.1 (1.1)	0
Number of Markets within 500m Buffer (Mean ± SD)	0.2 (0.5)	0
Distance to Nearest Selected Industries (m) (Mean ± SD)	937.4 (608.1)	0
Density of All Registered Roads of Montevideo (1km Buffer) (Mean ± SD)	15.3 (4.3)	0
Density of All Registered Roads of Montevideo (500m Buffer) (Mean ± SD)	14.0 (4.1)	0
Distance to Nearest Bus Route of Montevideo (m) (Mean ± SD)	143.6 (129.0)	0
Density of Bus Routes (1km Buffer) (Mean ± SD)	13.6 (12.1)	0
Density of Bus Routes (500m Buffer) (Mean ± SD)	26.9 (30.2)	0
Distance to Nearest Gas Station (m) (Mean ± SD)	1,110.1 (574.9)	0
Density of Gas Stations (1km Buffer) (Mean ± SD)	0.8 (1.0)	0
Density of Gas Stations (500m Buffer) (Mean ± SD)	0.2 (0.4)	0
Distance to Nearest Waterway (m) (Mean ± SD)	360.0 (446.2)	0
Density of Waterways (1km Buffer) (Mean ± SD)	1.0 (0.6)	0
Distance to Nearest River (m) (Mean ± SD)	1,573.8 (1,084.6)	0
Distance to Nearest Waste Disposal Container in Montevideo (m) (Mean ± SD)	1,244.9 (1344.0)	0
Density of Waste Disposal Containers in Montevideo (1km Buffer) (Mean ± SD)	2.0 (2.4)	0
Density of Waste Disposal Containers in Montevideo (500m Buffer) (Mean ± SD)	0.4 (0.9)	0
Percentage of Dwellings in Census Segment with City Network Water Source (Mean ± SD)	0.8 (0.2)	0
Percentage of Dwellings in Census Segment with a Well Water Source (Mean ± SD)	0.002 (0.02)	0
Percentage of Dwellings in Census Segment with Stream or Other Water Source (Mean ± SD)	0.002 (0.004)	0
Percentage of Dwellings that Dispose Human Waste through City Network (Mean ± SD)	0.53 (0.34)	0
Percentage of Dwellings that Dispose Human Waste through Septic Tank (Mean ± SD)	0.27 (0.25)	0
Percentage of Dwellings that Dispose Human Waste into Stream or Other (Mean ± SD)	0.02 (0.04)	0
Percentage of Dwellings Comprised of Tenants	73.01 (46.98)	0

Note. SD – Standard Deviation; UYU – Uruguayan Peso; m – meters; km – kilometers

Table 2. Model Performance across all base classifiers and stacked ensemble in the training, validation, and test datasets

	Ensemble-I				Ensemble-N				Ensemble-All			
	AUC	Prec.	Sens.	Spec.	AUC	Prec.	Sens.	Spec.	AUC	Prec.	Sens.	Spec.
GLM												
Training	0.77	0.19	0.94	0.55	0.60	0.0	0.0	0.50	0.79	0.15	0.86	0.53
Validation	0.63	0.29	0.69	0.60	0.56	0.05	0.75	0.56	0.61	0.26	0.67	0.59
Test	0.61	0.19	0.94	0.55	0.49	0.0	0.50	0.0	0.57	0.15	0.86	0.53
RF												
Training	1.0	1.0	1.0	1.0	0.98	0.87	0.95	0.90	1.0	1.0	1.0	1.0
Validation	0.73	0.31	0.76	0.62	0.50	0.05	1.0	0.56	0.74	0.60	0.74	0.71
Test	0.72	0.44	0.83	0.62	0.53	0.11	0.69	0.51	0.73	0.52	0.87	0.65
GBM												
Training	0.86	0.66	0.86	0.76	0.77	0.55	0.68	0.74	0.86	0.65	0.89	0.76
Validation	0.77	0.56	0.80	0.71	0.50	0.10	0.75	0.57	0.74	0.63	0.74	0.73
Test	0.75	0.59	0.82	0.68	0.59	0.01	1.0	0.50	0.70	0.52	0.84	0.65
DNN												
Training	1.0	0.99	1.0	0.99	0.77	0.43	0.78	0.65	1.0	0.97	1.0	0.97
Validation	0.67	0.06	0.80	0.56	0.52	0.05	0.75	0.56	0.60	0.24	0.68	0.59
Test	0.68	0.32	0.86	0.58	0.49	0.03	1.0	0.50	0.61	0.24	0.73	0.54
Ensemble												
Training	0.98	0.97	0.91	0.97	0.79	0.73	0.72	0.76	1.0	1.0	0.99	1.0
Validation	0.76	0.53	0.79	0.69	0.50	0.05	0.75	0.56	0.74	0.52	0.80	0.69
Test	0.75	0.56	0.79	0.65	0.51	0.0	0.0	0.50	0.75	0.63	0.79	0.69

Figure 1. ROC curves for Ensemble-I, Ensemble-N, and Ensemble-All across base classifiers on the test dataset

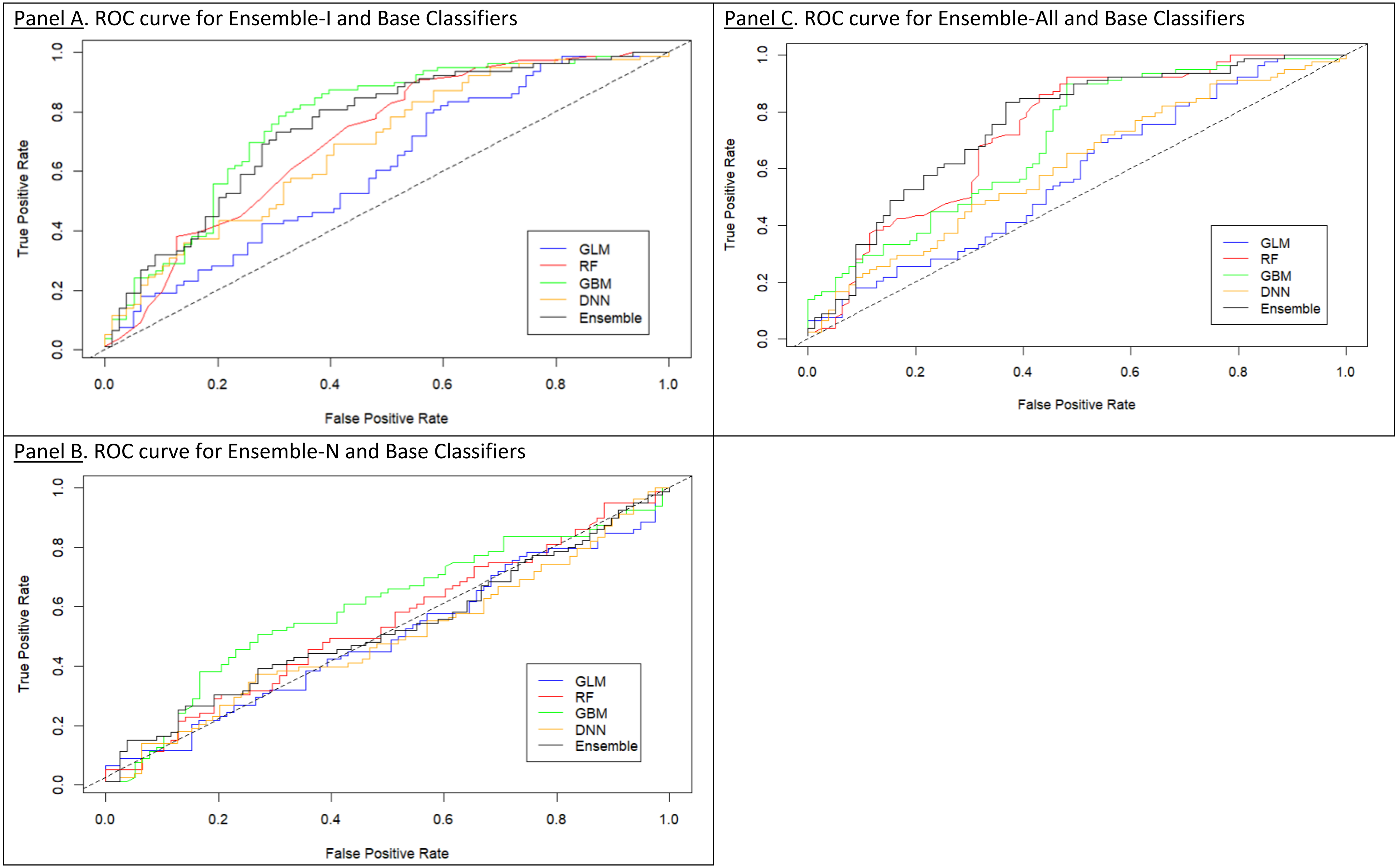

Figure 2. Variable Importance for Ensemble-I, Ensemble-N, and Ensemble-All (1-AUC) in the test dataset

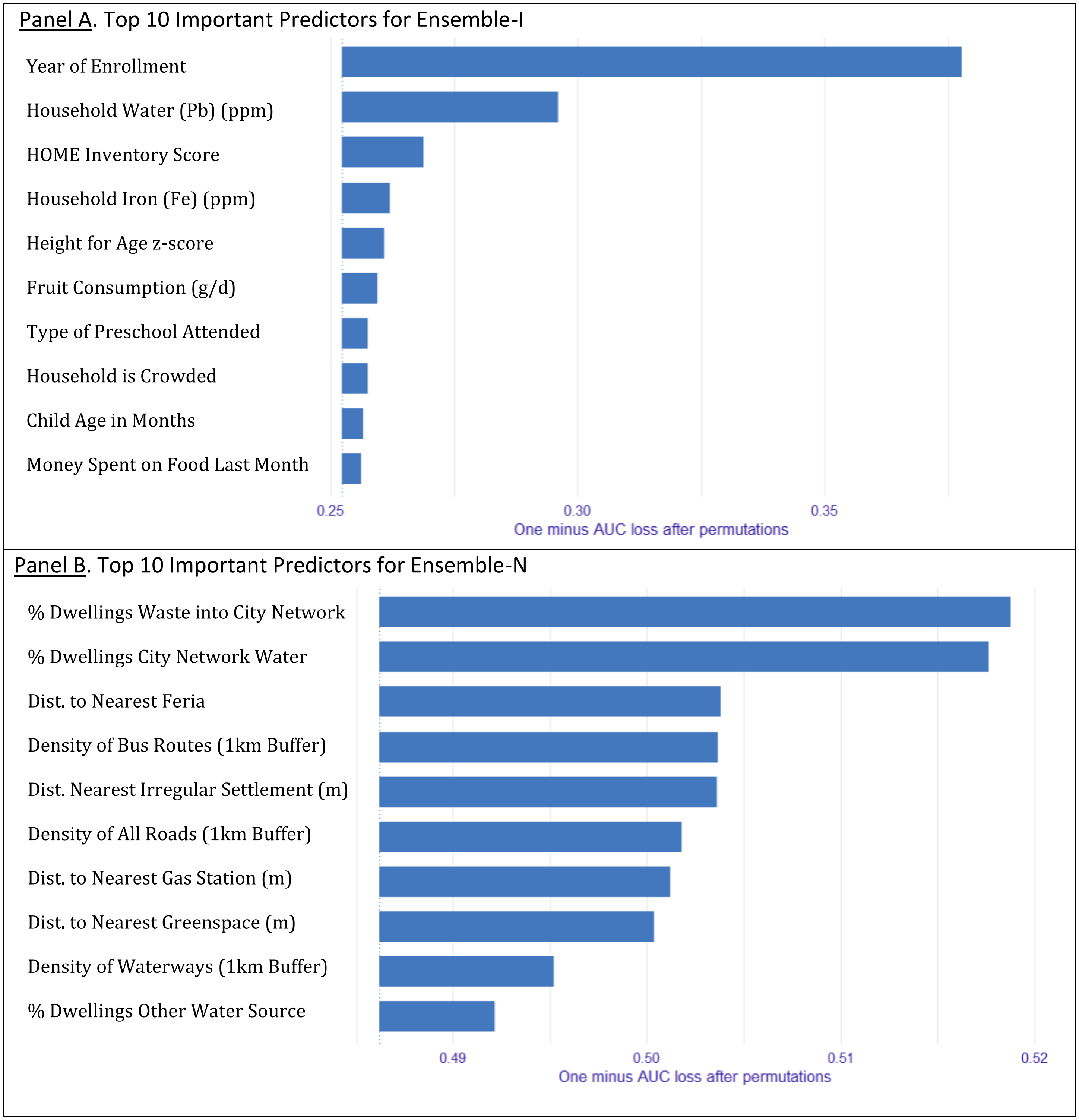

Panel C. Top 10 Important Predictors for Ensemble-All

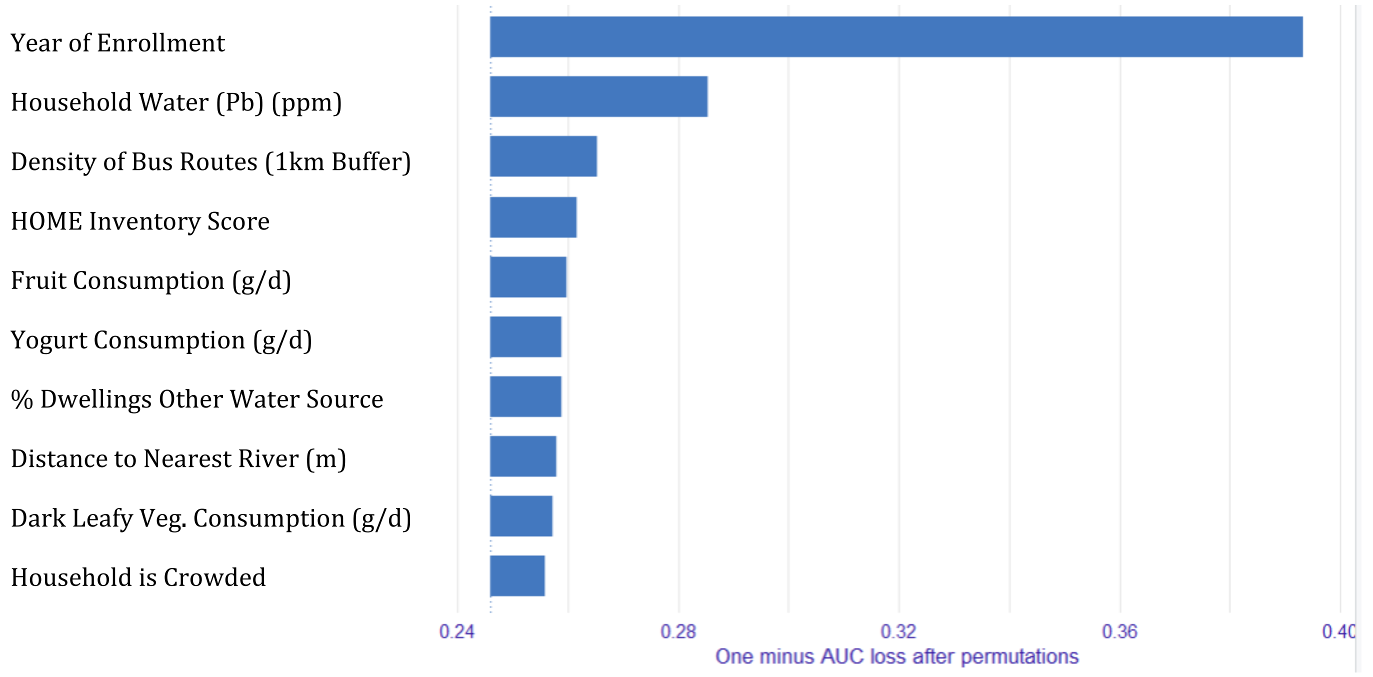

Statements and Declarations

Funding: The results reported herein correspond to specific aims of grant 1R01ES023423 to investigator Katarzyna Kordas from the National Institute of Environmental Health Sciences. This work was also supported by grant(s) R21ES019949 and 1R21ES16523 from the National Institute of Environmental Health Sciences (PI: Kordas).

Competing interests: No competing interests to declare.

Author Contributions**:** Conceptualization, S.F., and K.K.; methodology, S.F., Z.A., and G.U..; formal analysis, S.F..; resources, K.K., E.Q., N.M., and G.B.; data curation, S.F. E.Q., N.M., G.B., and K.K.; writing—original draft preparation, S.F.; writing—review and editing, S.F., K.K., C.C., Z.A., and G.U.; visualization, S.F. and Z.A.; supervision, K.K.; project administration, K.K. and E.Q..; funding acquisition, K.K. and E.Q.; All authors have read and agreed to the published version of the manuscript.

Availability of Data and Materials: Data and code may be available upon request.

Compliance with Ethical Standards

Competing interests: No competing interests to declare.

Research involving Human Participants and/or Animals: This research involves human subjects and not animals. The study protocols were approved by the institutional review boards of the University at Buffalo, the University of the Republic of Uruguay, and the Catholic University of Uruguay.

Informed Consent: All participants gave consent for participation and publication.

5.0 References

1. Pirkle JL, Brody DJ, Gunter EW, Kramer RA, Paschal DC, Flegal KM, et al. The decline in blood lead levels in the United States: the National Health and Nutrition Examination Surveys (NHANES). Jama. 1994;272(4):284-91.
2. Wheeler W, Brown MJ. Blood lead levels in children aged 1–5 years—United States, 1999–2010. MMWR Morbidity and mortality weekly report. 2013;62(13):245.
3. Egan KB, Cornwell CR, Courtney JG, Ettinger AS. Blood Lead Levels in US Children Ages 1–11 Years, 1976–2016. Environmental health perspectives. 2021;129(3):037003.
4. Rees N, Fuller R. The toxic truth: children's exposure to lead pollution undermines a generation of future potential. UNICEF; 2020. Report No.: 9280651404.
5. Canfield RL, Henderson Jr CR, Cory-Slechta DA, Cox C, Jusko TA, Lanphear BP. Intellectual impairment in children with blood lead concentrations below 10 µg per deciliter. New England journal of medicine. 2003;348(16):1517-26.
6. Lanphear BP, Hornung R, Khoury J, Yolton K, Baghurst P, Bellinger DC, et al. Low-level environmental lead exposure and children's intellectual function: an international pooled analysis. Environ Health Perspect. 2005;113(7):894-9.
7. Prevention CfDCa. CDC Updates Blood Lead Reference Value for Children 2021 [cited 2021. Available from: https://www.cdc.gov/media/releases/2021/p1028-blood-lead.html.
8. Lobo GP, Kalyan B, Gadgil AJ. Predicting childhood lead exposure at an aggregated level using machine learning. International Journal of Hygiene and Environmental Health. 2021;238:113862.
9. Kordas K, Ravenscroft J, Cao Y, McLean EV. Lead Exposure in Low and Middle-Income Countries: Perspectives and Lessons on Patterns, Injustices, Economics, and Politics. International journal of environmental research and public health. 2018;15(11):2351.
10. Cantor AG, Hendrickson R, Blazina I, Griffin J, Grusing S, McDonagh MS. Screening for elevated blood lead levels in childhood and pregnancy: updated evidence report and systematic review for the US Preventive Services Task Force. Jama. 2019;321(15):1510-26.
11. Potash E, Ghani R, Walsh J, Jorgensen E, Lohff C, Prachand N, et al. Validation of a Machine Learning Model to Predict Childhood Lead Poisoning. JAMA network open. 2020;3(9):e2012734-e.
12. Liu X, Taylor MP, Aelion CM, Dong C. Novel Application of Machine Learning Algorithms and Model-Agnostic Methods to Identify Factors Influencing Childhood Blood Lead Levels. Environmental Science & Technology. 2021.
13. Dong C, Taylor MP, Gulson B. A 25-year record of childhood blood lead exposure and its relationship to environmental sources. Environmental Research. 2020;186:109357.
14. Roy A, Queirolo E, Peregalli F, Mañay N, Martínez G, Kordas K. Association of blood lead levels with urinary F2-8α isoprostane and 8-hydroxy-2-deoxy-guanosine concentrations in first-grade Uruguayan children. Environmental research. 2015;140:127-35.
15. Parsons PJ, Chisolm J. The lead laboratory. Screening Young Children for Lead Poisoning: Guidance for State and Local Health Officials. 1997.
16. Ravenscroft J, Roy A, Queirolo EI, Mañay N, Martínez G, Peregalli F, et al. Drinking water lead, iron and zinc concentrations as predictors of blood lead levels and urinary lead excretion in school children from Montevideo, Uruguay. Chemosphere. 2018;212:694-704.
17. Kordas, Burganowski, Roy, Peregalli, Baccino, Barcia, et al. Nutritional status and diet as predictors of children's lead concentrations in blood and urine. Environment International. 2018;111:43-51.
18. Bradley RH, Caldwell BM, Corwyn RF. The Child Care HOME Inventories: Assessing the quality of family child care homes. Early Childhood Research Quarterly. 2003;18(3):294-309.
19. Bivand R, Rundel C, Pebesma E, Stuetz R, Hufthammer KO, Bivand MR. Package 'rgeos'. The Comprehensive R Archive Network (CRAN). 2017.

20. Bivand R, Keitt T, Rowlingson B, Pebesma E, Sumner M, Hijmans R, et al. Package 'rgdal'. Bindings for the Geospatial Data Abstraction Library Available online: https://cran r-project org/web/packages/rgdal/index html (accessed on 15 October 2017). 2015.
21. Pebesma E, Bivand R, Pebesma ME, RColorBrewer S, Collate A. Package 'sp'. The Comprehensive R Archive Network. 2012.
22. Leutner B, Horning N, Leutner MB. Package 'RStoolbox'. R Foundation for Statistical Computing, Version 01. 2017.
23. Hijmans RJ, Van Etten J, Cheng J, Mattiuzzi M, Sumner M, Greenberg JA, et al. Package 'raster'. R package. 2015;734.
24. Municipality of Montevideo Geographic Services. Sistema de Información Geográfica 2020 [Available from: http://sig.montevideo.gub.uy/.
25. Frndak S, Gallo Y, Queirolo EI, Barg G, Mañay N, Kordas K. A mixed methods study examining neighborhood disadvantage and childhood behavior problems in Montevideo, Uruguay. International Journal of Hygiene and Environmental Health. 2021;235:113753.
26. Marta S. Planet Imagery Product Specifications. Planet Labs: San Francisco, CA, USA. 2018:91.
27. Stekhoven DJ, Stekhoven MDJ. Package 'missForest'. 2012.
28. Stekhoven DJ. Using the missForest package. R package. 2011:1-11.
29. Tang F, Ishwaran H. Random Forest Missing Data Algorithms. Stat Anal Data Min. 2017;10(6):363-77.
30. James G, Witten D, Hastie T, Tibshirani R. An introduction to statistical learning: Springer; 2013.
31. Breiman L. Random forests. Machine learning. 2001;45(1):5-32.
32. Friedman JH. Greedy function approximation: a gradient boosting machine. Annals of statistics. 2001:1189-232.
33. Peterson LE. K-nearest neighbor. Scholarpedia. 2009;4(2):1883.
34. LeCun Y, Bengio Y, Hinton G. Deep learning. nature. 2015;521(7553):436-44.
35. Ahmed ZU, Sun K, Shelly M, Mu L. Explainable artificial intelligence (XAI) for exploring spatial variability of lung and bronchus cancer (LBC) mortality rates in the contiguous USA. Scientific Reports. 2021;11(1):24090.
36. Sesmero MP, Ledezma AI, Sanchis A. Generating ensembles of heterogeneous classifiers using stacked generalization. Wiley Interdisciplinary Reviews: Data Mining and Knowledge Discovery. 2015;5(1):21-34.
37. Candel A, Parmar V, LeDell E, Arora A. Deep learning with H2O. H2O ai Inc. 2016:1-21.
38. Biecek P. DALEX: explainers for complex predictive models in R. The Journal of Machine Learning Research. 2018;19(1):3245-9.
39. CEIC. Uruguay Exchange Rate against USD 2021 [Available from: https://www.ceicdata.com/en/indicator/uruguay/exchange-rate-against-usd.
40. Mahaffey KR, Annest JL, Roberts J, Murphy RS. National estimates of blood lead levels: United States, 1976–1980: association with selected demographic and socioeconomic factors. New England Journal of Medicine. 1982;307(10):573-9.
41. Moody HA, Darden JT, Pigozzi BW. The relationship of neighborhood socioeconomic differences and racial residential segregation to childhood blood lead levels in Metropolitan Detroit. Journal of Urban Health. 2016;93(5):820-39.
42. Desai G, Anzman-Frasca S, Vernarelli JA, Ravenscroft J, Yang J, Burstein G, et al. Examining Links Between Diet and Lead Exposure in Young Children: 2009 to 2014 National Health and Nutrition Examination Survey. Acad Pediatr. 2021;21(3):471-9.
43. Houston DK. Does vitamin C intake protect against lead toxicity? Nutrition reviews. 2000;58(3):73-5.

44. Ghanwat G, Patil AJ, Patil J, Kshirsagar M, Sontakke A, Ayachit RK. Effect of Vitamin C Supplementation on Blood Lead Level, Oxidative Stress and Antioxidant Status of Battery Manufacturing Workers of Western Maharashtra, India. J Clin Diagn Res. 2016;10(4):Bc08-11.

45. Hauptman M, Bruccoleri R, Woolf AD. An update on childhood lead poisoning. Clinical pediatric emergency medicine. 2017;18(3):181-92.

46. Mulhern R, Roostaei J, Schwetschenau S, Pruthi T, Campbell C, MacDonald Gibson J. A new approach to a legacy concern: Evaluating machine-learned Bayesian networks to predict childhood lead exposure risk from community water systems. Environmental Research. 2022;204:112146.

47. Goldstein A, Kapelner A, Bleich J, Pitkin E. Peeking Inside the Black Box: Visualizing Statistical Learning With Plots of Individual Conditional Expectation. Journal of Computational and Graphical Statistics. 2015;24(1):44-65.

48. Inglis A, Parnell A, Hurley CB. Visualizing Variable Importance and Variable Interaction Effects in Machine Learning Models. Journal of Computational and Graphical Statistics. 2022:1-13.

49. Mielke HW, Gonzales CR, Powell E, Jartun M, Mielke Jr PW. Nonlinear association between soil lead and blood lead of children in metropolitan New Orleans, Louisiana: 2000–2005. Science of the Total Environment. 2007;388(1-3):43-53.

50. Yeter D, Banks EC, Aschner M. Disparity in Risk Factor Severity for Early Childhood Blood Lead among Predominantly African-American Black Children: The 1999 to 2010 US NHANES. Int J Environ Res Public Health. 2020;17(5).
