# Supplementary material for: Predicting blood lead in Uruguayan children: Individual- vs neighborhood-level ensemble learners
Source: PLOS Glob Public Health. 2024 Sep 4;4(9):e0003607. doi: 10.1371/journal.pgph.0003607 (PMC11373808; doi:10.1371/journal.pgph.0003607)
Supplement: Attachment — Submitted filename: Reviewer Response - PLOS Global Public Health V2.docx [file pgph.0003607.s003.docx]

Reviewer Response – PLOS Global Public Health: Predicting Blood Lead in Uruguayan Children: Individual- vs Neighborhood-Level Ensemble Learners

Reviewer #1: The manuscript is well written and the study design is sound. Please find minor comments embedded in the manuscript test. The minor comments that I made are as follows:

*Response: We thank the reviewer for these kind comments.*

1. All acronyms/abbreviations should be defined in full at first use and used consistently thereafter. Please review throughout the manuscript.

*Response: Thank you for noticing this. We have made corrections to acronyms throughout the manuscript.*

2. 97 participants were excluded because of missing data. Were the available data for these excluded participants evaluated for homogeneity with those of included participants?

*Response: Thank you for pointing out this potential issue. There are differences in recruitment over time as the Ministry of Education allowed recruitment of children from public schools in 2015. This corresponded to the lowering of the analytical limit of detection to < 2. As a result, there are differences between those excluded and those included. However, the benefit of an LOD < 2 for scientific interest outweighs this limitation. We outline this starting on line 114.*

3. Different analysers were used for the analysis of BLL. Were results from these different analyzers evaluated for transferability?

*Response: Thank you for this comment. It is important to explain BLL testing and its evolution across the study vis-a-vis typical laboratory practice. At the start of the study, many blood samples were analyzed using flame Atomic Absorption Spectrometry (AAS), a standard method at the time that reflected the lead values of the population, which were still high. At this time, low sample volume necessitated a graphite furnace AAS. But as population lead levels began to fall, the laboratory made a switch to graphite furnace AAS and began refining its limits of detection to keep pace with the declining population levels. This is an accepted practice, and many analytical laboratories will continue to refine their limits of detection. For example, the limits of detection for lead changed three times in the US National Health Examination Survey from 1999 to 2010. To account for these and other changes in our study over time, we included in our predictive models an indicator variable for year of enrollment. In other analyses, not shown, we have found that year of enrollment was well correlated with a yes/no variable representing laboratory analytical methods. Our results in the present study also show that year of enrollment was a key predictor of children’s BLLs. It is also important to point out that the analytical laboratory that assessed BLLs in the study children participates in a number of inter-laboratory quality control programs and routinely analyzes standard/control samples to maintain high quality performance. These tests were included with each batch of samples and returned satisfactory results.*

4. The manuscript reports mean age as ~7years. One would have assumed that exact ages were recorded and the actual mean/median ages should be reported instead

*Response: Thank you, this was clarified on line 105*

5. Some of the data reported as mean±SD but the SD's are very large implying that the data might have been non normal and therefore needed to be reported as median(IQR). Could the authors please verify the distribution of their data and if non-parameteric use the appropriate summary statistics

*Response: Thank you for noticing this. We have changed non-normally distributed variables in Table 1 to reflect Median (Q1, Q3).*

6. The year of enrolment was reported to be a strong predictor of BLL. Weren't the years of enrollment also associated with different BLL measurement techniques? If that was the case, what impact would this have had on your findings?

*Response: Thank you for this point. Year of enrollment was an important predictor not only in our study but in others (Liu et al., 2021; Mulhern et al., 2022), and we believe that it reflects the secular trends in BLLs in the general population of children. Since submission of the present manuscript, we have published on the secular trends of blood lead levels in our study population. Implications of this research have been added to the discussion [line 415-17]. The importance of year of study in predictive models signals to public health officials wishing to try predictive models to identify at-risk groups or individuals that they must account for years of testing.* *It also suggests that the relevance of predictive models depends strongly on the age/recency of the data.* *Because predictive models require a large sample size, most studies will include data from multiple years. An indicator variable for year should be part of the predictors being tested, and future studies that have sufficiently large samples, may proceed by testing predictors in each study year separately to understand if other key predictors change over time.* *Our sample size did not allow us to perform such tests.*

7. Did your models also take into account the parent's occupation based on the possibility that parents could bring contaminated clothing from the work place etc?

*Response: Yes, we did include a variable that categorizes parental occupations that may be at risk for exposure: Table 1 - “Either Parent Works in a Job with Source of Exposure (% Yes)”*

8. Tables and figures should be self contained with all acronyms/abbreviations explained in full

*Response: Thank you and we have reviewed and added explanations for all acronyms throughout.*

Reviewer #2: I command the authors for doing this valuable research. There are a few changes/improvements that I would like to see before publishing this manuscript as follows:

*Response: Thank you for these comments.*

1. The authors should refrain from using active voices like I and we in the manuscript.

*Response: We agree and have corrected the active voices in the manuscript.*

2. Although the main aim of the work is related to prediction, I strongly recommend the authors elaborate more on the lead found in the sample of children involved. PLOS Global Public Health journal, as can be understood from the title, is concerned more in public health than in pure ML applications. If the authors published another paper on these results, summary of the results can be given in the current paper and that published paper should be referred to in this manuscript.

*Response: We have added additional information in the conclusions about the application of ML in predicting lead exposure for public health. Specifically, we urge the inclusion of predictors that encompass multiple proximal sources of lead exposure. Further development across years and contexts is also of importance.*

3. I suggest the authors add a couple of more recent references (after 2020) especially (but not necessarily) from PLOS journals.

*Response: We have included more recent and relevant publications in both the introduction and discussion. Thank you.*

4. "and evenly distributed by sex" Line 271 on page 7 should be "and almost evenly distributed by sex" or something similar to this statement. 52-48% is not exactly evenly distributed.

*Response: Thank you for noticing this. It has been corrected in this manuscript.*

5. The conclusions section is way too brief. The authors should elaborate more on the results in the conclusions section.

*Response: We have included additional discussion, cited newly published work, and emphasized the importance of proximal and culturally specific predictors of childhood lead exposure.*

---

PDF comments:

do metals such as iron, zinc, calcium, folate and vitamin C etc produce energy as implied by kilocalories?

*Response: Nutrients like iron, zinc, etc., do not contribute any calories. In our study, the intake of these nutrients is expressed per 1000 kcals to normalize their intake to calorie consumption. Each person consumes a different number of calories, contributed by a range of foods that may or may not be rich in nutrients. A person with a higher caloric intake has more opportunity to consume more of these beneficial nutrients. A person with lower caloric intake, on the other hand, may have less opportunity to consume these nutrients in foods. Therefore, comparing the nutrient intake of the two persons is only "apples-to-apples” when the nutrient intakes are normalized to calory intake. This is what we have done in our analyses and that is what our units represent.*
