# Supplementary material for: Predicting blood lead in Uruguayan children: Individual- vs neighborhood-level ensemble learners
Source: PLOS Glob Public Health. 2024 Sep 4;4(9):e0003607. doi: 10.1371/journal.pgph.0003607 (PMC11373808; doi:10.1371/journal.pgph.0003607)
Supplement: Attachment — Submitted filename: Comments to authors.docx [file pgph.0003607.s004.docx]

Comments to the Authors

Thank you for the opportunity to review this version of the manuscript. Most of the comments that I previously raised were satisfactorily addressed except for a some very minor comments:

1. On authors’ affiliations Lines 10-11, Department is spelts as ‘Departament’ in two instances.
2. Lines 272-282: I made a comment that the standard way to present means should mean±SD and I also highlighted that very large standard deviations tend to be associated with non-parametric data and in such cases, the mean and interquartile will be recommended as measures of central tendency. This was not addressed.
   a. Children had low to average BMI (M = 16.7, SD 273 = 2.6 kg/m2) (What does low to average mean in this case?)
   b. Children in our sample came from more disadvantaged neighborhoods (mean = 1.5, SD = 1.1) (What does the mean and SD refer to in this sentence?
   c. (mean = 143.6, SD = 129.0), within 400 meters of a waterway 280 (M = 360.0, SD =446.1), and within 1 kilometer of an industry using in toxic metals in production (M = 937.4, 281 SD = 608.1). Children also lived, on average, within 300 meters of greenspace (M = 246.3, SD = 208.4) (These Standard deviations are very high implying non-normal data. It is normally recommended to present such data as median(IQR)
3. In Table 1:
   a. Two variables do not have any data entered HAZ and WAZ. Please consider removing these if the data are not there.
   b. Hemoglobin is not measured in serum but whole blood. I suggest the serum prefixed to hemoglobin be removed.
4. Except for ‘Departament’ all these comments were made during the previous review cycle
