# Supplementary material for: Predicting blood lead in Uruguayan children: Individual- vs neighborhood-level ensemble learners
Source: PLOS Glob Public Health. 2024 Sep 4;4(9):e0003607. doi: 10.1371/journal.pgph.0003607 (PMC11373808; doi:10.1371/journal.pgph.0003607)
Supplement: Attachment — Submitted filename: Comments to authors_response.docx [file pgph.0003607.s005.docx]

Comments to the Authors

Thank you for the opportunity to review this version of the manuscript. Most of the comments that I previously raised were satisfactorily addressed except for a some very minor comments:

*Response: Thank you for agreeing to review the manuscript again.*

1. On authors’ affiliations Lines 10-11, Department is spelts as ‘Departament’ in two instances.

*Response: Thank you for noticing this typographical error. We have corrected to Department.*

2. Lines 272-282: I made a comment that the standard way to present means should mean±SD and I also highlighted that very large standard deviations tend to be associated with non-parametric data and in such cases, the mean and interquartile will be recommended as measures of central tendency. This was not addressed.

*Response: We thank the reviewer for allowing correction of these central tendency statistics in the main text.*

a. Children had low to average BMI (M = 16.7, SD 273 = 2.6 kg/m2) (What does low to average mean in this case?)

*Response: We changed this wording to: “Children had an average BMI of 16.7 (SD 2.6 kg/m2)”*

b. Children in our sample came from more disadvantaged neighborhoods (mean = 1.5, SD = 1.1) (What does the mean and SD refer to in this sentence?

*Response: This is a neighborhood disadvantage factor score. The methods for creating this factor score are cited in ‘Frndak S, Gallo Y, Queirolo EI, Barg G, Mañay N, Kordas K. A mixed methods study examining neighborhood disadvantage and childhood behavior problems in Montevideo, Uruguay. International Journal of Hygiene and Environmental Health. 2021;235:113753.’ We clarify that the mean of the factor for the city is 0. However, our sample has a mean score of 1.5 indicating that they are coming from areas of a higher disadvantage factor score. To clarify this sentence was changed to: “Children in SAM came from more disadvantaged neighborhoods (1.5 ± 1.1), compared to the overall city mean neighborhood disadvantage score of 0”*

c. (mean = 143.6, SD = 129.0), within 400 meters of a waterway 280 (M = 360.0, SD =446.1), and within 1 kilometer of an industry using in toxic metals in production (M = 937.4, 281 SD = 608.1). Children also lived, on average, within 300 meters of greenspace (M = 246.3, SD = 208.4) (These Standard deviations are very high implying non-normal data. It is normally recommended to present such data as median(IQR)

*Response: Thank you for the opportunity to make these corrections. We have reported Median and Interquartile ranges for this section.*

3. In Table 1:
   a. Two variables do not have any data entered HAZ and WAZ. Please consider removing these if the data are not there.

*Response: Thank you, we have added these statistics where needed.*

b. Hemoglobin is not measured in serum but whole blood. I suggest the serum prefixed to hemoglobin be removed.

*Response: Thank you for mentioning this. We have removed the serum prefix.*

4. Except for ‘Departament’ all these comments were made during the previous review cycle

*Response: Again, our sincere thanks for reviewing our manuscript.*
